# Section Plane Effects on Morphometric Values of Microcomputed Tomography

**DOI:** 10.1155/2019/7905404

**Published:** 2019-01-17

**Authors:** Young-Seok Park

**Affiliations:** Associate Professor, Department of Oral Anatomy, Dental Research Institute and Seoul National University School of Dentistry, Seoul, Republic of Korea

## Abstract

**Objectives:**

Histomorphometry is the established gold standard for inspection of trabecular microstructures in biomaterial research. However, microcomputed tomography can provide images from the perspective of various section planes. The aim of the present study was to evaluate the effects of different section planes, which may cause bias in two-dimensional morphometry, on the morphometric values of microcomputed tomography.

**Methods:**

A socket preservation technique was performed on the extracted premolar area of 4 beagle dogs. After an 8-week healing period, a total of 16 specimens were obtained and analyzed with conventional histomorphometry and microtomographic morphometry. Using the original images of the histologic specimens for comparison, the most similar tomographic image was selected by trial and error. Then, the section plane was then moved with ±79 *μ*m parallel offsets and rotated ±10° around the center from the occlusal view. The images were compared in terms of bone, graft, and noncalcified area, and the concordance correlation coefficient (CCC) was calculated.

**Results:**

There was a high CCC in the comparison between histomorphometric images and the most similar microtomographic images. However, the CCC value was low in the comparisons with both parallel movement and rotation. Our results demonstrate that the sectioning plane has a significant effect on measurements.

**Conclusion:**

Two-dimensional morphometric values for biomaterial research should be interpreted with caution, and the simultaneous use of complementary 3-dimensional tools is recommended.

## 1. Introduction

The internal microstructure of bone has been a subject of interest in various fields and particularly in dentistry [1]. Since the discovery of osseointegration, bone healing patterns around implant surfaces have been used as a primary means of evaluating prognosis [2, 3]. The increased demand for treatment in compromised situations requires excellent bone graft materials together with reliable techniques, and histologic confirmation is typically used to verify the results [4].

Histomorphometry (HM) can provide quantitative values of trabecular morphologic characteristics and therefore has been widely used for comparative biomedical research [5]. Trabecular morphologies have traditionally been evaluated two-dimensionally, with the structural parameters either inspected or measured from only selected sections of specimens [6]. Although this method has the advantages of high spatial resolution and image contrast, it is considered tedious, time consuming, and expensive [7]. Specimen preparation is typically laborious, involving embedding the specimen in resin followed by sectioning into thin slices. Another substantial disadvantage is the destructive nature of the procedure, which prevents specimens from being used for additional measurements, including other observation techniques and precludes analyses in different planes [3]. Several researchers have reported the use of three-dimensional (3D) histomorphometry on the basis of stereology, which requires an even greater amount of specimen preparation [2, 8–10]. This method is currently considered, as it were, “the gold standard”.

Microcomputed tomography (MCT) is a miniaturized version of computerized axial tomography and has recently been introduced to characterize 3D structures in bone tissue [11]. It provides precise measurements within a relatively short time without irreversible specimen preparation [11]. A key advantage of MCT is the ability to quantify 3D microstructure [12]. The final 3D product offered by MCT is a volume of scalar data which represent the test body through the attenuation coefficients [13]. The first attempt adapting the procedure of histomorphometry to digital images was presented by Feldkamp et al. [14]. They presented a method for quantification of bone microstructure using 5 parameters. The parameters are the 3D stereological indices which are extracted in line with the standard definitions used in histomorphometry: bone volume fraction (BV/TV), bone surface-to volume ratio (BS/BV), trabecular number (Tb.N), trabecular thickness (Tb.Th), and trabecular separation (Tb.Sp). There have been numerous reports regarding the MCT applications in various biomedical fields including dental research [15]. Recently,* in vivo* applications of MCT were introduced [16].

The low image qualities of MCT such as poor resolution and artefacts have remained as disadvantages compared to HM. Especially, the problems become more pronounced when metallic implants are nearby. However, nondestructive nature as well as providing 3D datasets makes MCT more and more popular in bone regeneration research in recent years [17]. Several studies designed to validate MCT as an alternative to HM have found the techniques to be comparable [2, 18–21], although these is some controversy [3, 22–24]. These studies have focused mainly on the feasibility of using MCT instead of the reference method of HM. A recent study attempted to combining 2D MCT onto 2D HM to quantify bone volume [25].

Bone is known to be highly anisotropic and the connectivity of trabeculae is important [26, 27], if possible, 3D investigations are highly encouraged [28]. However, simple two-dimensional (2D) HM has most frequently been adopted in the biomaterial literature instead of 3D stereologic HM [29–33]. Although the authors of those reports stated that they carefully selected the histologic specimens, it could be considered questionable whether or not these 2D data are truly representative of the entire specimen. Until now HM measurements, with or without other observations, have often been considered to provide conclusive evidence in biomaterial research even though they provide information from limited planes [23]. The objective of the present study was to evaluate the effects of different section planes on the morphometric value of microtomography using comparisons between different section planes as well as comparisons with histomorphometry.

## 2. Material and Methods

### 2.1. Animals

Four male beagle dogs (average age, 1.3 years; average weight, 10.3 kg) received dental prophylaxis to maintain periodontal health. Animal experiments were carried out in compliance with guidelines of the Institutional Animal Care and Use Committee of Seoul National University. The animals were acclimated before initiation of the experimental procedures and were housed individually in stainless steel cages kept in purpose-designed rooms that were air conditioned with 10–20 air changes/hour. Temperature and relative humidity were monitored daily and kept at 21±4°C and 50–60%, respectively, with a 12-h/12-h light/dark cycle. The animals were provided with access to water* ad libitum* and a standard laboratory diet.

### 2.2. Surgical Procedures

All surgical procedures were performed under general and local anesthesia induced by intravenous injection of atropine (0.04 mg/kg) and intramuscular injection of 2% xylazine hydrochloride (Bayer Korea Ltd, Seoul, Korea) and ketamine hydrochloride (Yuhan, Seoul, Korea). Routine dental infiltration anesthesia with 2% lidocaine hydrochloride/epinephrine 1:100,000 (Kwangmyung Pharmaceutical, Seoul, Korea) was used at the surgical site. Mandibular second and fourth premolars were extracted after elevation of the mucoperiosteal flap and the designated treatment was performed. Socket preservation procedures were performed using porcine hydroxyapatite grafts and polytetrafluoroethylene (PTFE) (Purgo, Seongnam, Korea). Primary wound closure was secured with interrupted single and mattress sutures using resorbable materials (Vicryl 5.0; Ethicon, Somerville, NJ, USA).

### 2.3. Postsurgical Procedures and Animal Sacrifice

For postsurgical care, the experimental dogs were administered 20 mg/kg cefazolin sodium (Yuhan) and a soft diet was provided. Plaque control was performed with topical application of chlorhexidine gluconate (0.12%) solution. After 8 weeks, the animals were sedated and euthanized with an overdose of sodium pentobarbital (100 mg/kg) for preparation of mandibular block sections. A total of 16 block biopsy specimens were collected.

### 2.4. Microcomputed Tomography

Specimen fixation was performed immediately after collection in 10% buffered formalin for 10 days. Then, they were scanned with a SkyScan 1172 micro-CT machine (Bruker-microCT, Kontich, Belgium) in wet scanning condition. The specimens were wrapped in parafilm (SERVA Electrophoresis, Heidelberg, Germany) to prevent drying and consequent distortion during the long scanning procedure. The system consisted of a sealed X-ray tube (20–100 kV/100 *μ*A) with a 2-*μ*m spot size and a precision object manipulator with two translations and one rotation direction. The system also included a 12-bit digital-cooled CCD camera (1024 × 1024 pixels) with fiber optics. Transmission of X-ray images was acquired from 600 rotation views through 180 degrees of rotation (rotation step=0.3) using a 0.5-mm aluminum filter to block low energy X-ray. Source acceleration voltage and current were set as 100 kV and 100 *μ*A, respectively. The exposure time was 316 ms for optimized clarity. All constructed cross sections contained 1,024 pixels, with a cross-section pixel size of 19.75 *μ*m.

### 2.5. Preparation of Histologic Specimens

Blocks from mandibles were sequentially dehydrated in 70% to 100% ethanol and then embedded in methacrylate (Technovit 7200 VCL; Kulzer, Wehrheim, Germany) and sectioned in the mesiodistal plane using a diamond saw (Exakt, Exakt Apparatebau, Norderstedt, Germany). The sections from the center of the entire block were reduced to a final thickness of 30 *μ*m and surface stained with hematoxylin-eosin.

### 2.6. Histomorphometric Analysis

Histologic analysis of the bone samples was performed using a light microscope with varying magnification (Olympus, Tokyo, Japan) connected to a computer. Digital images were captured and a computer-based image analysis system (Image J; National Institutes of Health, Bethesda, MD, USA) was used to quantify findings. A 4 × 4 mm^2^ corresponding area of interest was selected in the central region of the alveolar socket. The bone area, graft area, and noncalcified area were demarcated and segregated manually.

One examiner (Y-S P.) performed all histomorphometric analyses in a blinded and calibrated manner.

### 2.7. Morphometric Analysis of Microcomputed Tomographic Images

**Figure 1:**
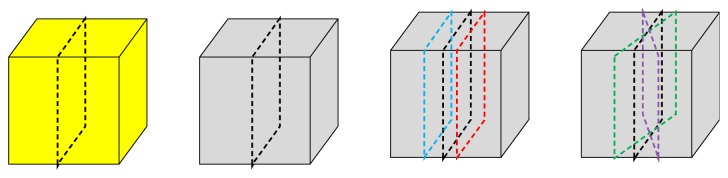
Diagram of different section planes. From the left, the section planes of histomorphometric (HM) image, the original microtomgraphy (MCT) image, offset MCT image, and rotation MCT image.

Image reconstruction was done using NRecon software (ver. 1.6.8). The image data (Image matrix 480 × 480 × 636) were reconstructed using a modified Feldkamp algorithm resulting in a reconstructed voxel size of 0.2 mm^3^. For comparison with the histomorphometric measurements, the MCT image that was the most similar to the original histologic image was chosen manually by adjusting the sectioning plane of 3D reconstructed data with parallel and angular movement. This procedure was basically trial and error to find the image closest to the original HM image. Morphometric measurement was performed with this selected image, which was called the original MCT image, after demarcating the bone, graft, and noncalcified areas in the same manner as for histomorphometry. Additionally, a total of 4, offset and rotated images were acquired to assess agreement between the original HM image and the original MCT image. In the present study, the offset image was defined as the 4th image when the section plane moved in a parallel manner. Therefore, the offset image was 79 *μ*m (19.75 × 4) from the original image, considering the thickness of cross-section pixel size. The rotated image was defined as the image that was rotated 10° around the center axis of the original image from the occlusal view. A ‘+' was used for designation of buccal parallel movement and clockwise rotation, and ‘-' was used for lingual parallel movement and counterclockwise rotation (Figure 1). To test the reliability of the measurements, 4 HM images and 8 MCT images were randomly selected and measured again on separate days 2 months after the initial measurement. To distinguish original bone, graft, and noncalcified areas, the selected gray scale images were segmented according to selected luminance thresholds. It should be noted that there were areas where the borderline between the 3 parameters were not clear and manual designations were necessary.

### 2.8. Statistical Analysis

**Figure 2:**
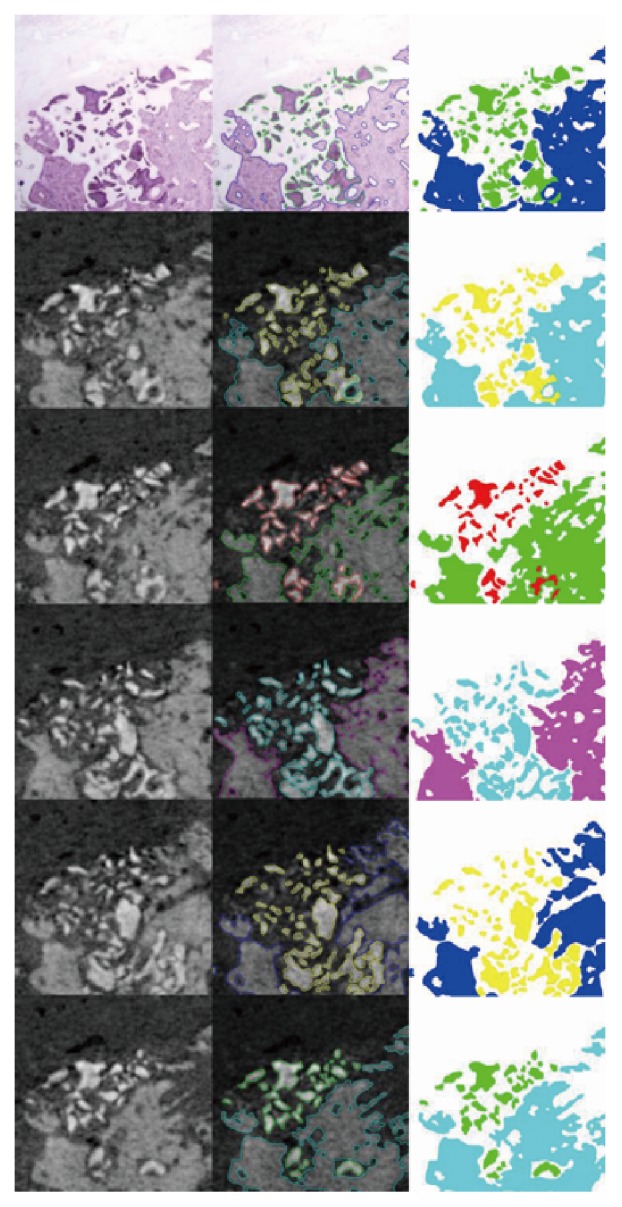
Example images of HM and morphometry of MCT. From top to bottom, the first row: HM image; the second row: the selected MCT image that is the closest possible to the HM (the original MCT); the third row: + 4 offset image from the original MCT; the fourth row: - 4 offset image from the original MCT; + 10 degree rotation image from the original MCT; - 10 degree rotation image from the original MCT. Left column: raw images; center column: segmented images into 3 regions, i.e., bone area, graft area and noncalcified area; right column: color-filled segmented images. The two different colors are used for this purpose at the images of middle and right column in the same row for discrimination. For example, at the image of middle column and first row of histomorphometric (HM) images, the green line demarcates the graft area whereas the blue line does the bone area. Then, the demarcated areas are filled with the same colors at the images of the right column and first row. Likewise, in the second row, yellow color is used for graft area and skyblue color is used for bone area. The differences occurring according to the section plane are easily identified.

**Figure 3:**
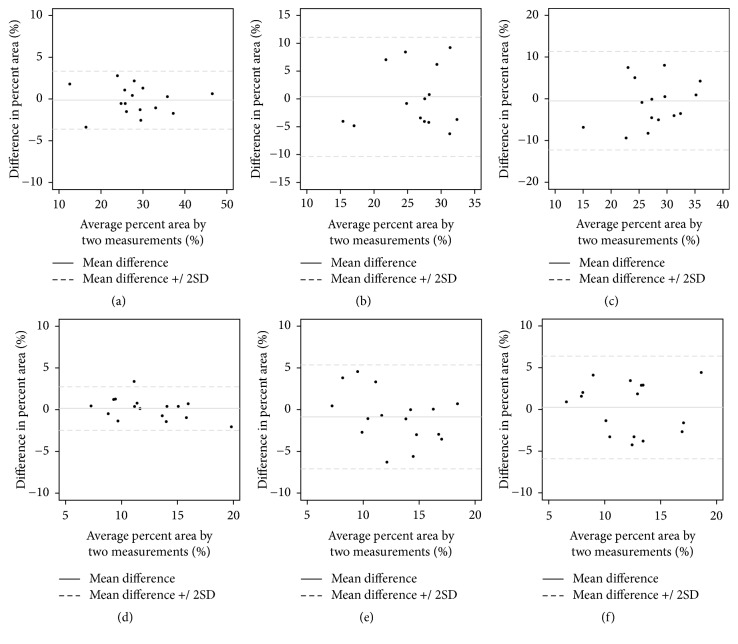
Examples of Bland-Altman plot of the measurements. (a) HM versus original MCT in bone area; (b) HM versus – 4 offset CT in bone area; (c) original MCT versus -10 degree rotation MCT in bone area; (d) HM versus original MCT in graft area; (e) HM versus + 4 offset CT in graft area; (f) original MCT versus + 10 degree rotation MCT. In contrast to the comparison between HM and original MCT, the other comparisons showed scattered and wider range of differences in percent area.

Interexaminer reliability in selection of the best-matched image of MCT to HM was checked in three sets of data. Quantitative morphometric data from MCT and HM were calculated as percent area of entire image (Figure 2). To evaluate the agreement between the measurements of 2 images, the concordance correlation coefficient (CCC) was obtained with mean difference of measurements and its standard deviation. The comparisons were performed with respect to percent area of bone and percent area of graft in 2 ways: HM value versus all the 5 morphometric values of MCT, and morphometric value of the original MCT versus the 4 remaining morphometric values of MCT from a different section plane. Bland-Altman plots were constructed to visualize the differences between the images (Figure 3). All analyses were conducted using the free statistical software R (version 3.0.1 R Core Team, 2013, Vienna, Austria).

## 3. Results

The interexaminer reliability coefficients in selection of best matching images ranged from 0.923 to 0.968. They were calculated for confirming the best matching MCT images to HM images selected by different examiners and by trial and error are reasonably similar at least in terms of morphometric value, i.e., area. The intraexaminer reliability coefficients ranged from 0.964 to 0.989. In terms of root mean squares, the random errors of estimation were lower than the value of 0.035 mm^2^ observed for the area measurements. There were no statistically significant differences between the test-retest measurements for any of the variables. This means the difference in the measurements of the same inspectors observed at different times are statistically insignificant.

The agreement between the original HM images and original MCT images was quite high in terms of the CCC of the measurements. The values of CCC were 0.976 (range, 0.934 to 0.992) for graft area and 0.924 (0.815 to 0.970) for bone area (Tables 1 and 2). This means that the best matching MCT images chosen by trial and error are nearly similar to the original HM images. In other words, 2D MCT images acquired at the similar section plane to the HM have the nearly identical morphologic features to the HM images. However, in comparisons between the value of the HM and the other 4 morphometric values of MCT, i.e., offset or rotated images, the CCC values plummeted with a wide range of limits in all cases of section plane movements, showing great standard deviation values of measurement differences (Figure 3). The CCC values for comparison area ranged from 0.607 to 0.814 in bone area and from 0.553 to 0.739 in graft area. The comparison between the original MCT images and offset or rotated MCT images also revealed low values of CCC, ranging from 0.569 to 0.788 in bone area and from 0.670 to 0.748 in graft area. Those results can be explained as the difference of section planes affects the morphometric values between MCT images acquired from different section planes. In addition, it is easily inferred that the HM images of different section planes, even of the minor differences, could have quite different morphometric values, although the comparison between HM was impossible in the present study due to the destructive nature of the methodology.

## 4. Discussion

Assessment of bone microarchitecture* in vivo* helps us understand whether grafted materials are successfully integrated into the host bone. Moreover, bone tissue microarchitecture is one parameter influencing “bone quality,” which is in itself a concept of debate that warrants further evaluation but is thought to be related to clinical success of implant survival on the grafted bone [3]. For quantitative investigation of structures, HM has been regarded as a reference technique. In particular, stereology-based HM can provide 3D structural information and is considered the gold standard [21, 34]. However, many studies have instead adopted simpler 2D HM for biomaterial-treated bone defects, most likely for practical reasons [29–31]. Except for the selected planes, it is impossible to get information from other perspectives in 2D HM. Thus, when using 2D HM the degree of sample isotropy or the representativeness of selected planes can be an important factor when interpreting the results. In addition to the substantial time and cost required even for the 2D technique, the necessary specimen preparation does not allow for any subsequent mechanical testing or secondary measurement. There can also be distortions of specimens during preparation, and a nontrivial amount of tissue loss is inevitable during serial section procedures. Therefore, even in a study using stereology-based HM, the number of specimens is sometimes not sufficient to truly represent the entire 3D structure [8].

In contrast, there are no gaps in images of MCT thus overcoming some of the limitations of analysis of 2D histologic sections [35]. Researchers have used MCT to investigate 3D connectivity in the trabecular network [36]. The recently introduced synchrotron MCT technique can deliver additional important information regarding the degree of mineralization, which can be obtained only indirectly by biological and pathological cues using conventional HM [9, 37]. However, MCT still cannot be a definitive tool because it lacks the unique advantages of histology. More specifically, it does not detect alterations in bone remodeling or metabolism, such as the number of bone cells and amount of osteoid or resorption surfaces [38, 39]. Thus, it is not prudent to assert that the morphometry of MCT can be used alone in evaluation of graft success.

It is unclear whether direct comparison between morphometric values of MCT and HM is appropriate. Theoretically, their direct comparison is only justified when an exactly identical plane is chosen or the entire 3D structure is perfectly reconstructed in both methods. However, 2D HM can provide information from only one or a few selected planes and the measurements can change considerably when the plane is changed. Therefore, the validity of comparison between the two methods is largely dependent on the degree to which 2D HM can represent properties of the entire specimen on the assumption that 3D MCT can be comparable to the gold standard of 3D HM. Not a few studies have already shown that 3D MCT has potential as an alternative to conventional HM because it provides similar or corresponding morphometric values to those obtained from HM. However, 3D MCT morphometric values might be similar just by chance and it is natural that they would show linearity with 2D HM. Although we do not know what percentage of total 2D HMs represents the entire specimens well enough, it is very likely that there are some cases that they do not. Furthermore, there is a possibility of misinterpreting this characteristic when presenting study results. Therefore, the present study focused on the difference between 2D cuts from different section planes.

The results of our study showed that differences in section planes could result in substantial difference in morphometric values, at least in MCT images. By analogy, morphometric values of HM might also differ according to the change of section plane, although we could not verify this because the method precludes reinvestigation from another perspective, and especially from other angles.

The similarities between HM images and the original or the best matching MCT images in the present study were substantial but not perfect. Even though image selection was conducted carefully, it was very difficult to obtain totally identical images within the scale of micrometers. This led to inherent disagreement in measurement values from the two images. However, the disagreement was usually minimal and is line with the previous reports. Interestingly, the discrepancy became more remarkable in the concordance area rate, which evaluates how precisely the images overlap in the superimposition instead of providing a cumulative area comparison (data not presented).

In this study, agreement between images was investigated by calculating the CCC, which is an indication of agreement between two variables. The CCC was calculated instead of the commonly used intraclass correlation coefficient (ICC) because the ICC requires equal marginal distributions of the model. If the distributions are unequal or inaccurate there is a possibility that the ICC captures unreliable deviations [40, 41]. Unlike the ICC, the CCC can distinguish inaccuracy from unreliability [42]. In addition, the popularly used Pearson correlation coefficients, which were high in this study, have innate weakness because they show only the linearity between values for repeated measurements. Bland-Altman plots as presented in Figure 3 are widely used to evaluate agreement between two different measurement techniques. The mean difference depicted in the Y-axis is the estimated bias, and the standard deviation expresses the random fluctuations around the mean. The plots of comparison between best matching images (Figures 3(a) and (d)) showed more aggregated dots and smaller standard deviation compared with comparison between other combinations.

Additionally, there is a technical problem to be solved in MCT. The structural indices determined from the MCT measurements are dependent on the incorporated thresholding procedure [43]. Chappard et al. [11], as well as other authors, have warned that the high correlation between HM and MCT is influenced by the threshold options and 3D algorithm used [34, 44, 45]. To exclude ambiguity, we analyzed the area by segmenting it into three simple parameters: bone, graft, and noncalcified areas. Nonetheless, there were portions that were difficult to distinguish. This problem will likely be solved in the near future as device performance, including signal-to-noise ratio, improves.

In this regard, the present study has several limitations. Segmentation is very important to not only the identification but also the performance of quantified measurements. In addition, partial volumes could be a major concern which relates to binary segmentation problem. In the present investigation, neither automated approaches nor designated thresholds were used. The segmentation was done manually as in the conventional HM. Therefore, refinement of segmentation or implementing more sophisticated methodology may result in more elaborate discrepancies. Further studies are suggested using those enhanced technologies, with which there are several studies applying in various topics [6, 17, 46, 47]. Nevertheless, the results from this study warns that it is dangerous to depend entirely upon the HM acquired from a couple of planes without confirm by other methods.

On the basis of these findings, the morphometry of MCT might not be regarded as an alternative to HM, although the data acquisition was not performed by state-of-the-art fashion. However, it is evident that there are section plane effects on HM, which might cause bias or wrong interpretation of the experiment. Therefore, the MCT could be used as a complementary or it should be used as a confirmatory method for analyzing the success of bone grafts from the 3D perspective, since 2D HM images may not always be representatives of whole specimens. Further studies are recommended to investigate the histomorphometry of implant specimens, which are particularly difficult to prepare sequentially.

## 5. Conclusion

High concordance was found in comparisons of morphometric values between 2D HM images and 2D MCT images manually selected as most nearly identical. However, the results of the study indicated that changes in the section plane caused substantial differences in the morphometric value of the 2D MCT images. By analogy, it is possible that the different section planes also affect 2D HM morphometric values and any interpretations should be made with caution and with consideration of other evidence. Concurrent use of MCT or other 3D imaging modalities are recommended for 3D understanding of bone microstructures.

## Figures and Tables

**Table 1:** Concordance correlation coefficients of measurements in bone area.

Variables	CCC	Lower limit	Upper limit	Mean Δ ± SD
Comparison with HM				

Original MCT	0.976	0.934	0.992	0.121 ± 1.733
+ 4 offset MCT	0.814	0.596	0.920	-0.379 ± 5.338
– 4 offset MCT	0.735	0.408	0.895	-0.183 ± 5.777
+10 degree rotation MCT	0.607	0.230	0.826	0.565 ± 6.054
-10 degree rotation MCT	0.658	0.276	0.861	0.191 ± 7.298

Comparison with original MCT				

+ 4 offset MCT	0.788	0.541	0.910	-0.500 ± 5.389
– 4 offset MCT	0.732	0.403	0.893	-0.303 ± 6.167
+10 degree rotation MCT	0.633	0.274	0.837	0.944 ± 3.511
-10 degree rotation MCT	0.569	0.135	0.820	0.070 ± 8.217

**Table 2:** Concordance correlation coefficients of measurements in graft area.

Variables	CCC	Lower limit	Upper limit	Mean Δ ± SD
Comparison with HM				

Original MCT	0.924	0.815	0.970	-0.119 ± 1.305
+ 4 offset MCT	0.628	0.263	0.836	0.886 ± 3.106
– 4 offset MCT	0.739	0.481	0.879	0.594 ± 2.795
+10 degree rotation MCT	0.553	0.119	0.809	0.147 ± 3.319
-10 degree rotation MCT	0.630	0.239	0.845	-0.360 ± 3.013

Comparison with original MCT				

+ 4 offset MCT	0.748	0.442	0.898	1.006 ±2.645
– 4 offset MCT	0.811	0.578	0.922	0.713 ±2.471
+10 degree rotation MCT	0.682	0.303	0.875	0.267 ± 2.993
-10 degree rotation MCT	0.670	0.282	0.869	-0.241 ± 3.056

## Data Availability

The data used to support the findings of this study are available from the corresponding author upon request.
